# Metagenomic Sequencing From Mosquitoes in China Reveals a Variety of Insect and Human Viruses

**DOI:** 10.3389/fcimb.2018.00364

**Published:** 2018-10-19

**Authors:** Pengpeng Xiao, Chenghui Li, Ying Zhang, Jicheng Han, Xiaofang Guo, Lv Xie, Mingyao Tian, Yiquan Li, Maopeng Wang, Hao Liu, Jingqiang Ren, Hongning Zhou, Huijun Lu, Ningyi Jin

**Affiliations:** ^1^Institute of Military Veterinary, Academy of Military Medical Sciences, Changchun, China; ^2^Yanbian University Medical College, Yanji, China; ^3^Institute of Virology, Wenzhou University, Wenzhou, China; ^4^College of Veterinary Medicine, College of Animal Science, Jilin University, Changchun, China; ^5^Yunnan Institute of Parasitic Diseases, Simao, China; ^6^Jiangsu Co-innovation Center for Prevention and Control of Important Animal Infectious Diseases and Zoonoses, Yangzhou, China; ^7^School of Life Sciences and Engineering, Foshan University, Foshan, China; ^8^Division of Economic Animal Epidemic, Institute of Special Economic Animal and Plant Sciences, Changchun, China

**Keywords:** metagenomic analysis, mosquito, virome, virus detection, phylogenetic analysis

## Abstract

We collected 8,700 mosquitoes in three sites in China, which belonged to seven species. Their viromes were tested using metagenomic sequencing and bioinformatic analysis. The abundant viral sequences were detected and annotated belonging to more than 50 viral taxonomic families. The results were verified by PCR, followed by phylogenetic analysis. In the present study, we identified partial viral genes of dengue virus (DENV), a novel circovirus (CCV), densovirus (DNV), Japanese encephalitis virus (JEV), and Wuhan mosquito virus (WMV) in mosquitoes. Metagenomic analysis and PCR amplification revealed three DENV sequences, which were as homologous to the NS3 gene of DENV from Singapore isolated in 2005, with at least 91% nucleotide (nt) identity. Seven fragments of JEV encoding structural proteins were identified belonging to genotype I. They all shared high homology with structural protein genes of JEV isolated from Laos in 2009. The production of infectious virus particles of the newly isolated virus YunnanJEV2017-4 increased after passage from the BHK-21 cell line to the Vero cell line. Novel circovirus-related genes were identified and as being related to an unnamed gene of a mosquito circovirus (MCCV) sequence from the USA isolated in 2011, with at least 41% nt identity: this distant relationship suggests that the parent virus might belong to a novel circovirus genus. Additionally, numerous known viruses and some unknown viruses were also detected in mosquitoes from Yunnan province, China, which will be tested for propagation.

## Introduction

Mosquitoes are significant insect vectors for a variety of infectious viruses covering Japanese encephalitis virus (JEV), Circovirus (CCV), Zika virus (ZIKV), Dengue virus (DENV), and Densovirus (DNV), which pose a significant threat to human health and induce an economic burden worldwide (Pham et al., 2005; Klungthong et al., 2007). Mosquitoes are infected with viruses when consuming blood from hosts undergoing viremia. Then the viruses replicate and propagate in the insect host and are introduced into further victims during biting and blood feeding (Ritchie et al., 2007; Motooka et al., 2015). Mosquitoes feed on a wide range of hosts, including humans and other vertebrates, invertebrates, and plants (Shi et al., 2014). Yunnan province in China harbors a diverse range of mosquito-borne viruses (Feng et al., 2015); therefore, regional surveillance is imperative. The detection of viruses in mosquitoes is usually performed by reverse transcription polymerase chain reaction (RT-PCR) and nested PCR approaches (Almeida et al., 2005; Houghton, 2009; Li et al., 2015; Sim et al., 2015). However, compared with Illumina sequencing (Alquezar-Planas et al., 2013; Cholleti et al., 2016; Ergunay et al., 2017), these traditional methods are time-consuming and labor intensive to detect low-level viromes in mosquito vectors (He et al., 2013; Miesen et al., 2016). Thus, metagenomic analysis of mosquitoes is likely to be of great value to avoid missing the detection of viruses with high infectivity and pathogenicity in different collection sites, and the detection of previously unknown viruses.

The present study aimed to build a valid surveillance method to monitor the distribution of viruses from mosquitoes in Yunnan province, China and to provide useful insights into viral isolation, prevention, and control. Diverse and abundant viromes from mosquitoes isolated in Yunnan province were investigated using metagenomic sequencing and PCR. The presence of DENV, JEV, and novel circovirus were confirmed, and JEV was isolated. This preliminary exploration of the metagenomes of mosquito-borne viruses lays the foundation for further research on the territorial distribution, diversity, and surveillance of mosquito-borne viruses in China and other countries.

## Materials and methods

### Mosquito collection

We collected 8,700 living or freshly dead mosquitoes in Yunnan province, China, during September and October, 2017. The acquired mosquitoes were composed of *Culex tritaeniorhynchus* (*C. tritaeniorhynchus*), *Armigeres obturbans* (*Ar. obturbans*)*, Aedes albopictus* (*Ae. albopictus*), *Anopheles sinensis* (*An. sinensis*), *Anopheles maculatus* (*An. maculates*), *Anopheles mininus* (*An. minimus*), and *Culex quinquefasciatus* (*C. quinquefasciatus*). The three mosquito samples were grouped based on collection sites (Table 1). The species of mosquitoes in all groups were identified separately and stored at −80°C (Supplementary Table 1). This research was approved by the Ethics Committee and the Research Ethics Committee of Yanbian University Medical College. All experiments involved in active viruses were performed in Biological safety protection third-level laboratory.

**Table 1:** Mosquito samples employed in metagenomic analysis and data from Illumina sequencing.

**Sample**	**Species**	**Number**	**Location**	**Total reads number**	**Average (nt)**	**Viral reads**	**Viral contigs**
Sample I	Mosquito Community^#^	1000	Ninger County^*^	8,618,693	192.20	1,032,569	4,627
Sample II	Mosquito community^#^	1000	Jinggu county^*^	8,797,151	194.59	763,217	3,819
Sample III	Mosquito community^#^	6700	Dali City^*^	8,786,675	192.34	1,263,176	6,573
Total/average				26,202,519	193.05	2,340,048	15,019

**Figure 1:**
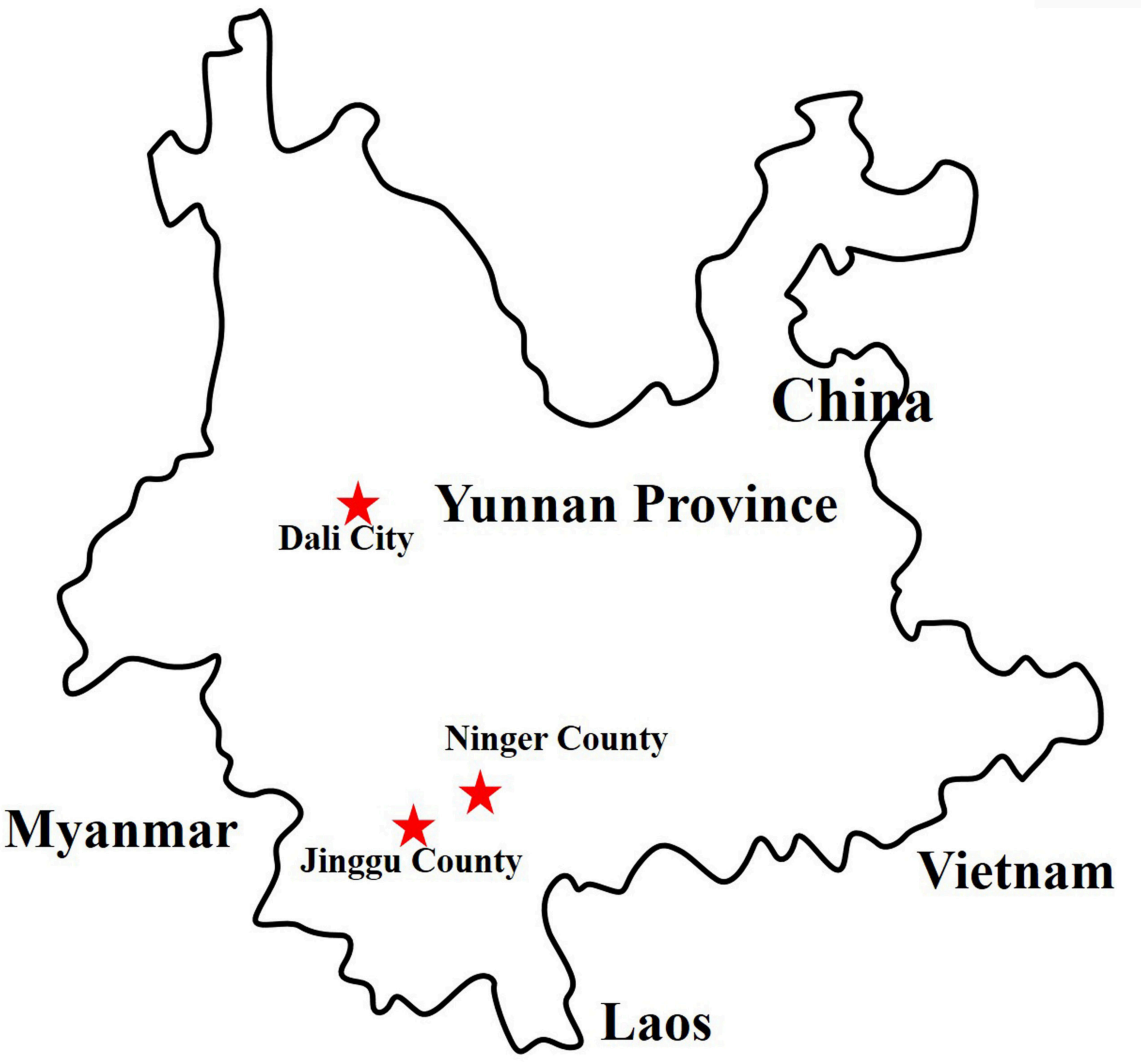
Distribution of sample collection sites in Yunnan province, China, 2017. The sample collection sites were labeled with red star.

* *Collection sites (Figure 1) were Ninger county (N 23° 03′, E 101° 02′) and Jinggu county (N 23° 50′, E 100° 71′) in Puer city, and Dali city (N 25° 69′, E 100° 19′). ^#^Mosquito species were consist of C. tritaeniorhynchus, Ar. obturbans, Ae. albopictus, An. sinensis, An. maculates, An. Minimus, and C. quinquefasciatus*.

### Preparation of mosquito samples

Briefly, mosquitoes of three samples were mixed with SM buffer (50 mM Tris, 10 mM MgSO4, 0.1 M NaCl, pH 7.5) (MeilunBio, Dalian, China) respectively and ground using a tissue grinder. After removal of mosquito debris and other materials, the mixed samples were centrifuged at 13000 × *g* for 20 min, and the supernatants were employed to extract the viral nucleic acid.

### Extraction of viral nucleic acid and reverse transcription

Clearly, 14 U of Turbo DNase (Ambion, Austin, TX, USA), 25 U of Benzonase Nuclease (Novagen, San Diego, CA, USA), 20 U of RNase I (Fermentas, Ontario, Canada), and 10 × DNase buffer (Ambion) were added to 127 μL of the supernatants to a final volume of 150 μL, followed by digestion at 37°C for 1 h. After removing free nucleic acid and eliminating the contaminating host genomic DNA, the viral nucleic acid in the obtained products was extracted using a Virus Nucleic Acid Kit (Bioer Technology, Hangzhou, China) according to the manufacturer's instructions. The total viral nucleic acids were reverse transcribed using anchored random primers and Superscript III reverse transcriptase (Invitrogen, Carlsbad, CA). Anchored random primers (Table 2) were added separately to the viral nucleic acid and incubated at 75°C for 5 min and placed on ice for 5 min for denaturation. To acquire the reverse transcribed product, 40 U of RNase OUT, 200 U of SuperScript III reverse transcriptase, 1 μL of 0.1 M dithiothreitol (DTT), 1 μL of 10 mM dNTPs, 4 μL of 5 × first strand buffer, and RNase-free H_2_O were added to a final volume of 20 μL. It was incubated at 25°C for 10 min followed by 50°C for 60 min, and 75°C for 10 min.

**Table 2:** Barcode DNA employed in metagenomic analysis (He et al., 2013).

**Primer type**	**Primer number**	**Primers (5^′^-3^′^)**
Anchored Random Primers	RT1	GCCGGAGCTCTGCAGATATCNNNNNN
	RT 2	GTATCGCTGGACACTGGACCNNNNNN
	RT 3	ATCGTCGTCGTAGGCTGCTCNNNNNN
	RT 4	CGTAGATAAGCGGTCGGCTCNNNNNN
	RT 5	CATCACATAGGCGTCCGCTGNNNNNN
	RT 6	CGCAGGACCTCTGATACAGGNNNNNN
	RT 7	CGTCCAGGCACAATCCAGTCNNNNNN
	RT 8	CCGAGGTTCAAGCGAGGTTGNNNNNN
	RT 9	ACGGTGTGTTACCGACGTCCNNNNNN
	RT 10	CGACCCTCTTATCGTGACGGNNNNNN
	RT 11	GAGCCCCTAGACACAACGACNNNNNN
	RT 12	GGTGGGCGTGTGAAATCGACNNNNNN
	RT 13	GAAAATGAGAGGGGAGGCGGNNNNNN
Barcode primers	Primer1	GCCGGAGCTCTGCAGATATC
	Primer2	GTATCGCTGGACACTGGACC
	Primer3	ATCGTCGTCGTAGGCTGCTC
	Primer4	CGTAGATAAGCGGTCGGCTC
	Primer5	CATCACATAGGCGTCCGCTG
	Primer6	CGCAGGACCTCTGATACAGG
	Primer7	CGTCCAGGCACAATCCAGTC
	Primer8	CCGAGGTTCAAGCGAGGTTG
	Primer9	ACGGTGTGTTACCGACGTCC
	Primer10	CGACCCTCTTATCGTGACGG
	Primer11	GAGCCCCTAGACACAACGAC
	Primer12	GGTGGGCGTGTGAAATCGAC
	Primer13	GAAAATGAGAGGGGAGGCGG

### Synthesis of double-strand cDNA (dscDNA)

To degrade the free RNA, 1 μL of RNaseH was added to the above-obtained products. To synthesis dscDNA, anchored random primers were added and incubated at 75°C for 5 min and on ice for 5 min for denaturation. Then 1 μL of Klenow fragment, 1 μL of 10 mM dNTPs, 2 μL of 10 × Klenow buffer, and 6 μL of dd H_2_O were added. Subsequently, the samples were incubated at 37°C for 60 min, followed by 75°C for 10 min. To eliminate phosphates and free single-strand nucleic acid in the dscDNA reaction, 0.5 μL of Exonuclease I, 1 μL of shrimp alkaline phosphatase (SAP, Takara, Dalian, China), 5 μL of 10 × phosphatase buffer, and 24 μL of DEPC H_2_O were added and incubated at 37°C for 60 min followed by 75°C for 10 min.

### Sequence-independent single primer amplification (SISPA) and purification of PCR products

To obtain large quantity of the viral nucleic acids, the dscDNA was amplified using SISPA. The 50-μL reaction comprised 10 μL of dscDNA, 2 μL of barcode primer (Table 2), 1 μL of Accuprime Taq DNA polymerase, 5 μL of 10 × Accuprime buffer I, and 32 μL of dd H_2_O. The PCR condition comprised 95°C for 3 min; 40 cycles of 95°C for 20 s, 54°C for 20 s, and 68°C for 70 s, with a final extension at 68°C for 7 min. The acquired PCR products were purified using a PCR purification kit (QIAGEN, Hilden, Germany) and eluted in 30 μL of TE buffer (100 mM Tris-HCl, 10 mM EDTA, pH8.0).

### Metaviral sequencing

The purified PCR products were sent to the Wuhan Genome Institute (BGI, Shenzhen, China) for Illumina sequencing. Concisely, to obtain ~180 bp DNA fragments, the purified PCR products were ultrasonicated and dATP and Klenow fragment were added to produce 3′-dA overhangs. To establish genomic DNA libraries, DNA fragments were bound to Illumina adaptors and amplified using PCR with adaptor primers. Amplicons were ligated to flow cells to which fluorescently labeled dNTPs were added. Identification of DNA sequences was performed by the Sequencing-by-Synthesis method (SBS, Illumina). Base calling was monitored by the program GAPipeline (BGI) with default settings. No-calling reads and adaptor sequences were eliminated. The remaining sequences were assembled into contigs using SOAPdenovo software (BGI). Contigs and sequences longer than 100 bp were defined as significant data for further *in silico* analysis.

### Computational analysis

Alignments of contigs and sequences were operated using BLASTx and BLASTn with the non-redundant and viral reference databases in GenBank (https://www.ncbi.nlm.nih.gov/genbank/). BLAST hits with an *E* value ≤ 10e^−5^ were considered significant. After elimination of bacterial and eukaryotic sequences, virus-like sequences were analyzed.

### Identification of detected viruses

In accordance with the alignment results of viral contigs and the match position of viral contigs with the corresponding viruses in GenBank, specific primers (Table 3) were designed and synthesized to identify the detected viruses. Viral nucleic acids were extracted using a Virus Nucleic Acid Kit (Bioer Technology) and amplified using the designed primers and a PCR Master Mix (Tiangen, Beijing, China).

**Table 3:** Primer pairs used in PCR identification.

**Primer Name**	**Primers (5^′^-3^′^)**	**Product (bp)**
YunnanWMV8-2017-1/2/3/4/5-F	GTGAAGAAAGATGAAGGAT	561
YunnanWMV8-2017-1/2/3/4/5-R	CTATCCAGGGCCTCCCATCA	
YunnanDENV2017-1/2/3-F	AGGAGCCCTGTGGGACGTCCC	1836
YunnanDENV2017-1/2/3-R	TTTTCTTCCACTGGCAAACTCCTT	
YunnanDNV2017-1/2/3-F	ACTGGACCAACCGTTGGTG	387
YunnanDNV2017-1/2/3-R	AGGGCTATGTGCGTTAACAAT	
YunnanDNV2017-4/5-F	ACAAAACAAACTCATCAGTCGGC	429
YunnanDNV2017-4/5-R	TTAGATGATGTAAGGGTTTTGGTTG	
YunnanDNV2017-6/7/8-F	CTACTAGCAATGGTTAAACTGG	801
YunnanDNV2017-6/7/8-R	TCAAGAATCCGGCTGTTTGGTA	
YunnanMCCV2017-1/2-F	GCCGGAGCTCTGCAGATAT	645
YunnanMCCV2017-1/2-R	CCGGAGCTCAGACGTGTGCT	
YunnanJEV2017-1/2-F	AGCCGGAGCTCTGCAGATAT	870
YunnanJEV2017-1/2-R	CTACAGACGTGTGCTCTTC	
YunnanJEV2017-3-F	TTTAACTGTCTGGGAATGG	1064
YunnanJEV2017-3-R	ACCAACCTCCCCACAGGGG	
YunnanJEV2017-4/5/6/7-F	ATGAAGCTATCAAACTTTCAAG	2001
YunnanJEV2017-4/5/6/7-R	GGCATGCACATTGGTCGCT	
JEV-F^*^	CTATTGGTCGCTCCGGCTTACAGT	1500
JEV-R^*^	TGTCAATGGCGCAGCCAGTGTC	

* *The primers used in JEV identification after viral isolation*.

### Phylogenetic analysis

The acquired PCR products were sequenced, and the sequences were aligned against sequences of representative viruses using Clustal W version 2.0. (Accession numbers are shown in the phylogenetic trees). Maximum-likelihood phylogenetic trees were produced using MEGA 7 with 1,000 bootstrap replicates.

### Cell culture

The hamster cell line BHK-21 and Vero cell line (both available in our laboratory) were employed in this study. BHK-21 cells and Vero cells were cultured in Dulbecco's modified Eagle's medium (DMEM. HyClone, Logan, UT, USA) with 10% fetal bovine serum (FBS. HyClone) and 1% penicillin and streptomycin (Pen Strep. HyClone), and incubated at 37°C in 5% CO_2_.

### Isolation of viruses

Viral isolation was conducted for the positive samples. Briefly, the mosquito samples were diluted 7-fold with DMEM containing 2% FBS and the infected BHK-21 cells were cultured for 5–7 days. Cultures were examined daily for evidence of a virally induced cytopathic effect (CPE). Cultures without a CPE were blind passaged three times.

### Identification of the isolated virus by PCR and western blot

The isolated virus YunnanJEV2017-4 was identified by PCR technique using the specific primers (Table 3) of the envelope (E) protein genes. Moreover, the further verification was performed by Western blot method with an anti-E monoclonal antibody (Abcam, Cambridge, UK) as primary antibody and a HRP-conjugated goat anti-mouse antibody (ZSGB-Bio, Beijing, China) as second antibody.

### Observation of negative-stain electron microscopy

BHK-21 cells with suspected JEV-induced CPE were collected by repeated freezing and thawing three times, followed by centrifugation at 10,000 × *g* for 15 min. The obtained virus suspension was centrifuged at 60,000 × *g* for 4 h and the supernatant was removed gently and discarded. Subsequently, the viral precipitate was resuspended with an isometric mixture of 6.1% (v / v) pH 7.2 glutaraldehyde (HEDEBIO, Beijing, China) fixative and DMEM, of which 25 μL were added to the copper grid. After desiccation, one drop of 3% phosphotungstic acid (JINDU, Shanghai, China) was added for negative staining. Before observation under an electron microscopic(FEI, Hillsboro, USA), the grid was placed in an incubator (SANYO, Osaka, Japan) at 37°C for desiccation.

### Detection of the viral titer of different passages

The viral titers of different passages of JEV (passage 5 and 10) were evaluated in BHK-21 cells in triplicate. Cells were infected with serial tenfold dilutions of JEV and incubated for 120 h at 37°C. The cytopathic effect of JEV at each dilution was evaluated in eight replicates. The TCID_50_ (50% tissue culture infective dose) was calculated by the Reed-Muench method (Krah, 1991).

### The variability of envelope (E) genes of different passage viruses

The obtained JEV (P5 and P10) from BHK-21 cells line were passaged in Vero cells line respectively. Viral RNA of JEV (P5 and P10) from infected BHK-21 and Vero cells was extracted from the supernatant using an RNA viral kit (Bioer Technology). The cDNA was synthesized with the purified RNA using a SuperScript™ III first-strand synthesis system (Invitrogen) with the reverse primer JWR1 5′-AGATCCTGTGTTCTTCCTCACCACCA-3′, according to the manufacturer's instructions. Envelope genes of JEV (P5 and P10) from infected BHK-21 and Vero cells were amplified using the paired primers shown in Table 3 and sequenced. The alignment of the four envelope genes was performed using MEGA 7.0.

### Statistical analysis

The data analysis was performed using SPSS 17.0 software. Group comparisons for viral titers were done by *t*-test for independent means. Experiments were performed in triplicate and a minimum of three independent experiments were evaluated. The value of *P* < 0.05 was considered statistically significant.

GenBank accession numbers

Amplified sequences were deposited in the GenBank under the following accession numbers: YunnanWMV8-2017-1/2/3/4/5 (MH193505–MH193509), YunnanDENV2017-1/2/3 (MH193510–MH193512), YunnanDNV2017-1/2/3/4/5/6/7/8 (MH193513–MH193520), YunnanMCCV2017-1/2 (MH193521–MH193522), YunnanJEV2017-1/2/3/4/5/6/7 (MH193523–MH193529), respectively.

## Results

### Metaviral sequencing and the virome of mosquitoes

**Figure 2:**
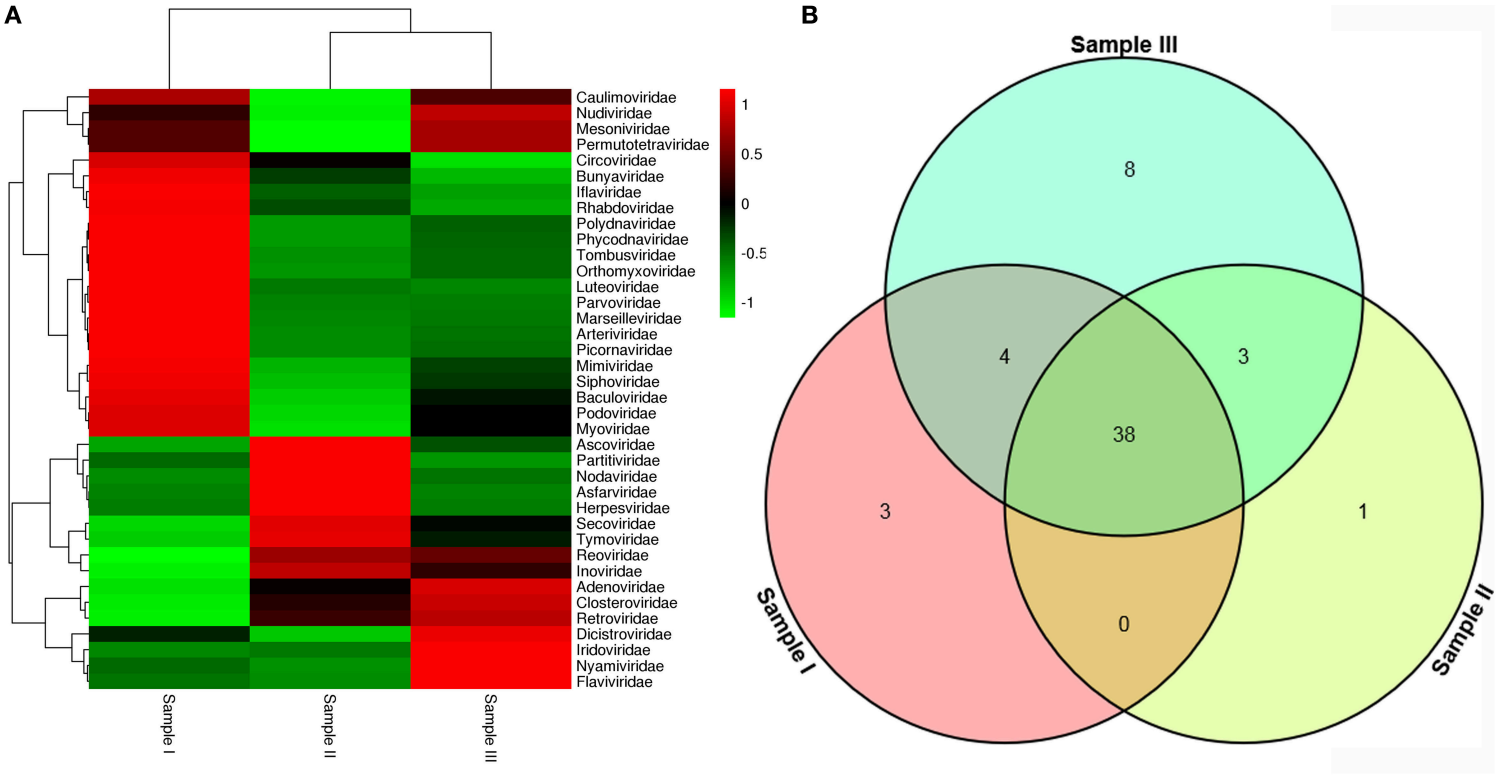
Abundance of viral families and their relationship in three samples. Viral sequences were sorted according to viral families and the relative abundance were displayed in Heat Map **(A)**. The amount of viral families in each sample and their intersection among the three samples were shown in Venn diagram **(B)**.

To obtain the clean data of the virome in mosquitoes, contaminating host sequences and barcode DNA were eliminated. A total of 26,202,519 reads were acquired by Illumina sequencing, with averaging read lengths of 193.05 nt (Table 1). The amount of viral sequences was 11.98, 8.68, and 14.38% in sample I, II, and III respectively, of which the relative abundance of viral families was shown in Figure 2A. The intersection among the three samples was displayed in Figure 2B. There were 45, 42, and 53 viral families in sample I, II, and III respectively. Remarkably, the three samples all possessed representative sequences from 38 common families: *Luteoviridae, Marseilleviridae, Tombusviridae, Parvoviridae, Adenoviridae, Baculoviridae, Nudiviridae, Podoviridae, Reoviridae, Secoviridae, Bunyaviridae, Iridoviridae, Iflaviridae, Dicistroviridae, Ascoviridae, Inoviridae, Orthomyxoviridae, Asfarviridae, Caulimoviridae, Nodaviridae, Herpesviridae, Arteriviridae, Circoviridae, Rhabdoviridae, Mimiviridae, Closteroviridae, Polydnaviridae, Phycodnaviridae, Picornaviridae, Partitiviridae, Retroviridae, Myoviridae, Nyamiviridae, Mesoniviridae, Permutotetraviridae, Siphoviridae, Flaviviridae, Tymoviridae* (Supplementary Table 2).

### PCR verification of the metavirome results

Viral sequences were assembled into contigs using SOAPdenovo. From the 15,019 assembled viral contigs, it was detected that 46 WMV8-like contigs with a read coverage of 43 × (278–836 nt), 83 DENV-like contigs with a read coverage of 34 × (207–2356 nt), 164 DNV-like contigs with a read coverage of 127 × (257–936 nt), 26 MCCV-like contigs with a read coverage of 17 × (219–1053 nt), and 182 JEV-like contigs with a read coverage of 139 × (317–3285 nt). The WMV8-like contigs shared 91.3–93.3% nt identity with known WMV8 sequences. The DENV-like contigs shared 91.9–92.8% nt identity with known DENV sequences. The DNV-like contigs shared 80.6–90.0% nt identity with known DNV sequences. The MCCV-like contigs shared 41.6–42.3% nt identity with known MCCV sequences. The JEV-like contigs shared 86.6–97.7% nt identity with known JEV sequences. To confirm the outcome of metavirome sequencing, specific primers were designed and synthesized to amplify the identified viruses (Table 3). The following were the results of PCR identification of detected viruses in mosquito samples.

#### PCR amplification of wuhan mosquito virus

**Figure 3:**
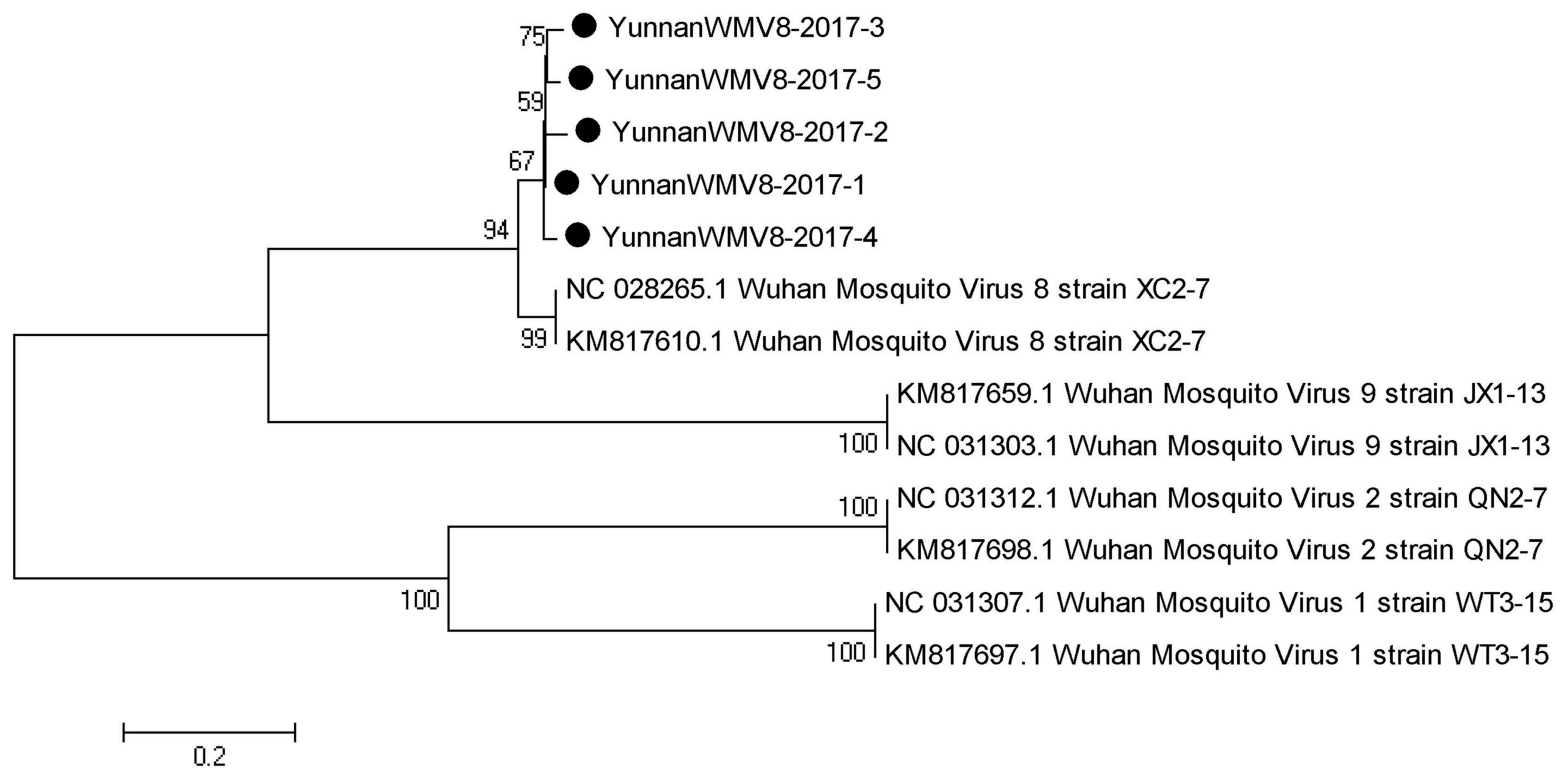
Phylogenetic tree of Wuhan mosquito virus. Phylogenetic trees based on RNA-dependent RNA polymerase (L) gene of Wuhan mosquito virus. Using the Maximum-likelihood method in MEGA 7.0 software. Bootstrap values were calculated with 1,000 replicates. Black solid circles indicate the genes identified in this study.

Based on the alignment results of viral contigs, viral PCR amplicons from sample I shared high identity with Wuhan mosquito virus (WMV) genes. Five 561-nucleotides-long segments (YunnanWMV8-2017-1/2/3/4/5) that shared ~96–98% nt identity and ~89–95% amino acid (aa) identity with each other were isolated. BLASTN against the GenBank identified these segments as homologous to the L gene of WMV, with at least 91% nt identity and 87% aa identity suggesting that viruses were from a WMV8 variant (Figure 3).

#### PCR amplification of dengue virus

**Figure 4:**
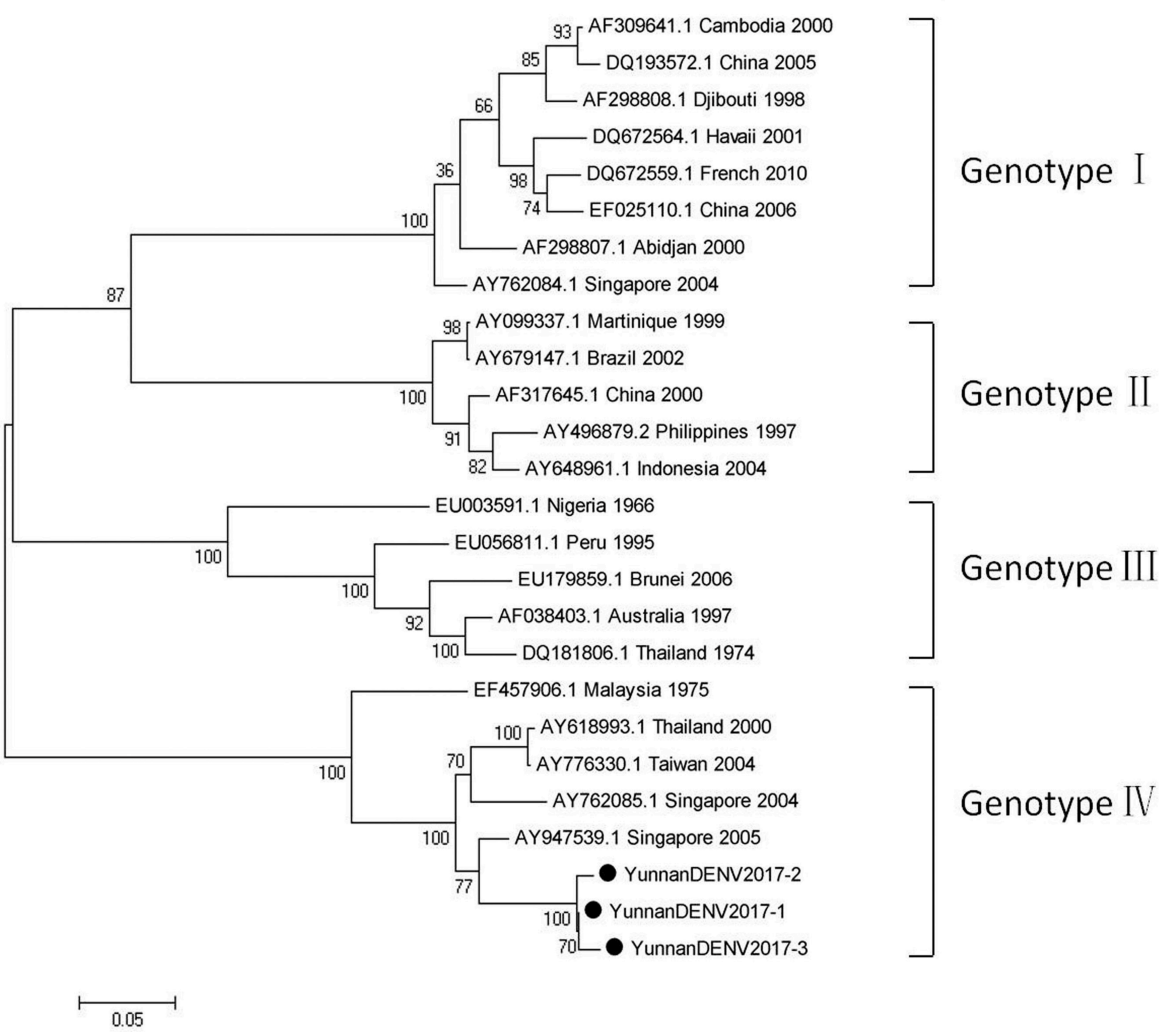
Phylogenetic tree of DENV. Phylogenetic tree based on Non-structural protein 3 (NS3) gene of DENV. Using the Maximum-likelihood method in MEGA 7.0 software. Bootstrap values were calculated with 1,000 replicates. Black solid circles indicate the genes identified in this study.

Based on the alignment results of viral contigs, viral PCR amplicons from samples III shared high identity with dengue virus (DENV, genus *Flavivirus*, family *Flaviviridae*). Three 1836-nucleotides-long segments (YunnanDENV2017-1/2/3) that shared ~98–99% nt identity and ~95–97% aa identity with each other were isolated. BLASTN against the GenBank identified these segments as homologous to the non-structural protein 3 (NS3) gene of DENV sequence from Singapore isolated in 2005, with at least 91% nt identity and 88% aa identity suggesting that viruses were from its variant (Figure 4). Phylogenetic analysis showed that the newly identified DENV sequences were genotype IV genes.

#### PCR amplification of mosquito densovirus

**Figure 5:**
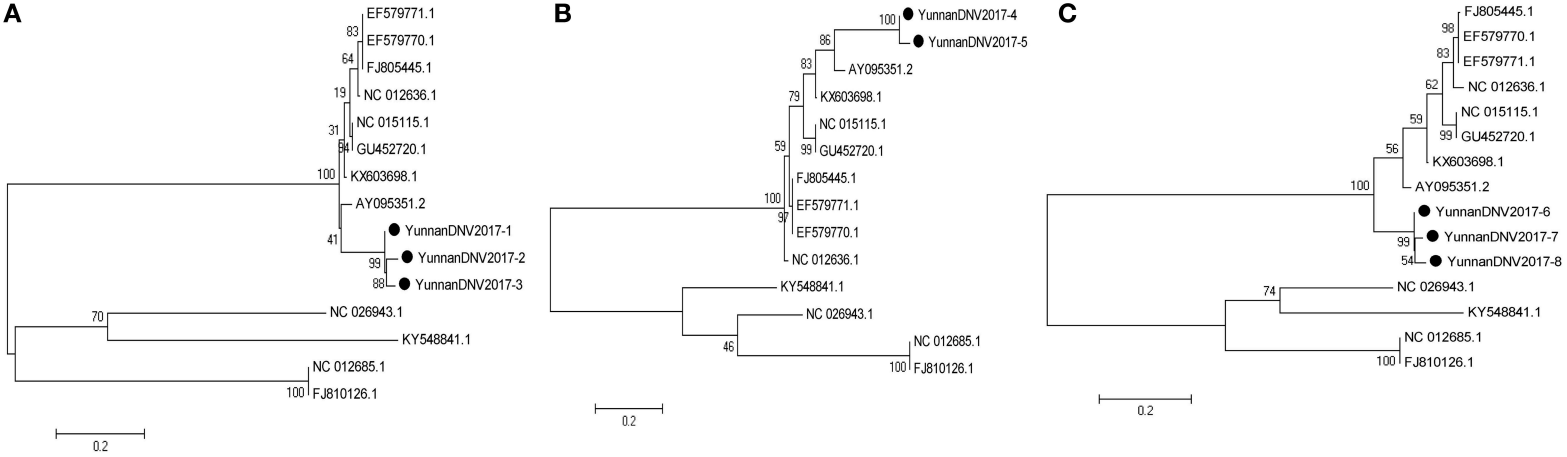
Phylogenetic trees of Mosquito densovirus. Phylogenetic tree based on Non-structural protein 2 (NS2) gene of Mosquito densovirus together with YunnanDNV2017-1/2/3 **(A)**, YunnanDNV2017-4/5 **(B)**, and YunnanDNV2017-6/7/8 **(C)**. Using the Maximum-likelihood method in MEGA 7.0 software. Bootstrap values were calculated with 1,000 replicates. Black solid circles indicate the genes identified in this study.

In line with the alignment results of viral contigs, viral PCR amplicons from sample II shared high identity with Aedes albopictus densovirus (DNV, genus *Brevidensovirus*, family *Parvoviridae*). Eight viral segments were isolated, among which three 387-nucleotides-long segments (YunnanDNV2017-1/2/3), two 429-nucleotides-long segments (YunnanDNV2017-4/5) and three 801-nucleotides-long segments (YunnanDNV2017-6/7/8) that shared ~96–97%, ~97% and ~96–98% nt identity and ~89–93%, ~93% and ~91–95% aa identity respectively with each other. BLASTN against the GenBank identified these segments as homologous to the non-structural protein 2 (NS2) gene of DNV from China isolated in 2006 (Figure 5), with at least 87, 80, and 83% nt identity and 85, 79, and 85% aa identity respectively.

#### PCR amplification of mosquito circovirus

**Figure 6:**
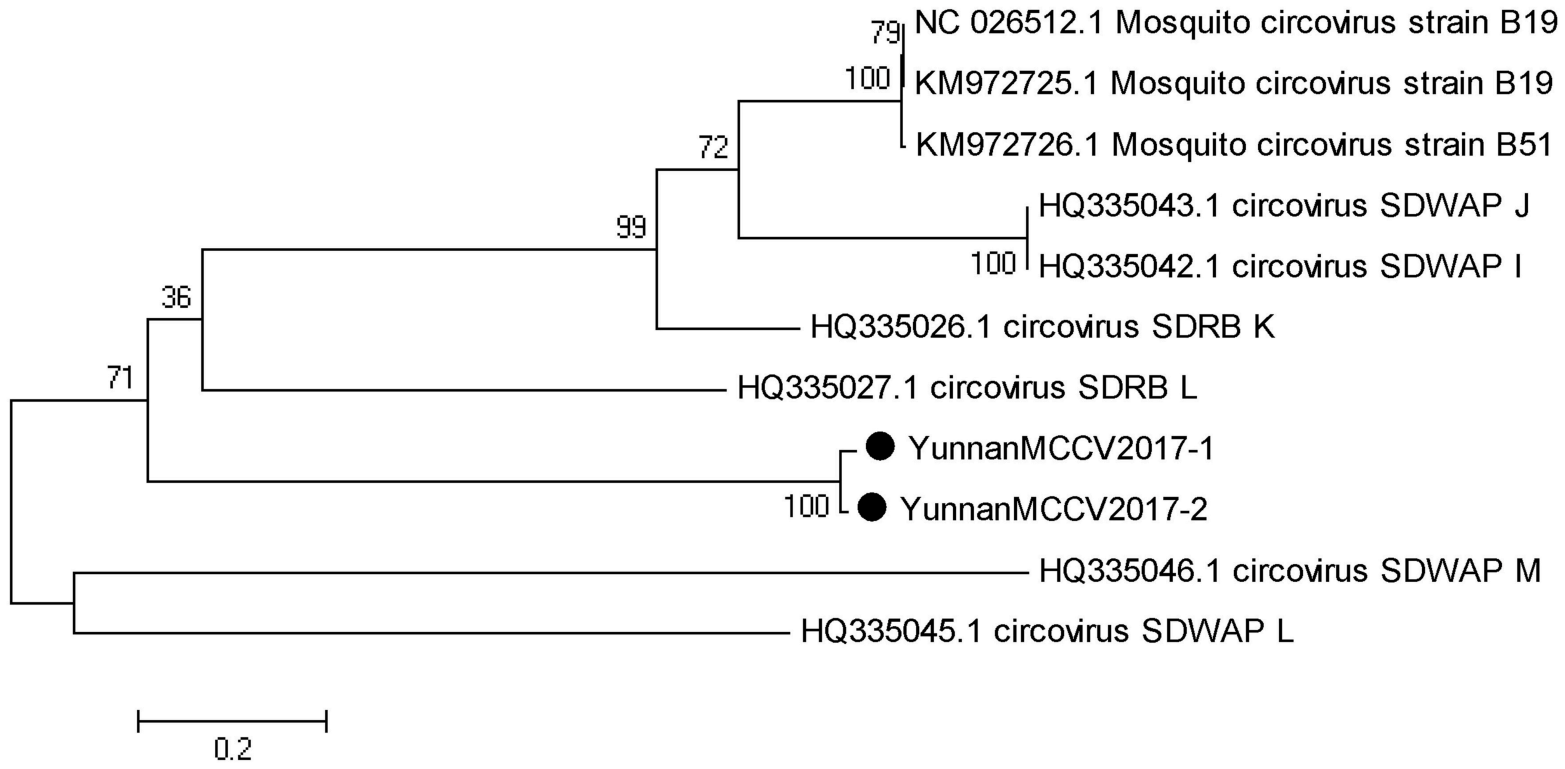
Phylogenetic tree of Mosquito circovirus. Phylogenetic tree based on Mosquito circovirus. Using the Maximum-likelihood method in MEGA 7.0 software. Bootstrap values were calculated with 1,000 replicates. Black solid circles indicate the genes identified in this study.

Based on the alignment results of viral contigs, viral PCR amplicons from samples III shared high identity with mosquito circovirus (MCCV, family *Circoviridae*). Two 645-nucleotides-long segments (YunnanMCCV2017-1/2) that shared ~97% nt identity and ~93% aa identity with each other were isolated. BLASTN against the GenBank identified these segments as homologous to an unnamed gene of MCCV, with at least 41% nt identity and 40% aa identity with MCCV sequence from America isolated in 2011 suggesting that viruses may be novel circovirus (Figure 6).

#### PCR amplification of japanese encephalitis virus

**Figure 7:**
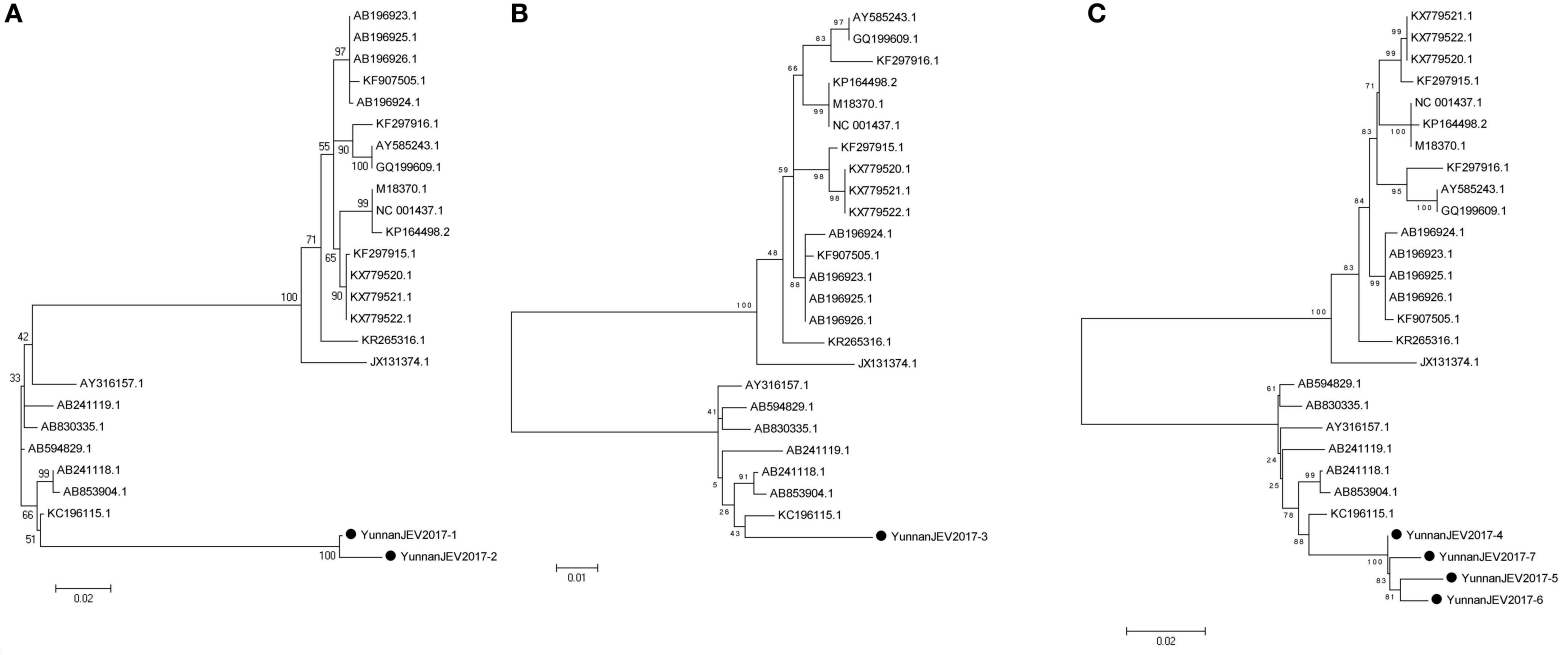
Phylogenetic trees of JEV. Phylogenetic tree based on C+prM gene of JEV together with YunnanJEV2017-1/2 **(A)**, E gene of JEV together with YunnanJEV2017-3 **(B)**, and prM+E gene with YunnanJEV2017-4/5/6/7 **(C)**. Using the Maximum-likelihood method in MEGA 7.0 software. Bootstrap values were calculated with 1,000 replicates. Black solid circles indicate the genes identified in this study.

According to the alignment results of viral contigs, viral PCR amplicons from sample III shared high identity with Japanese encephalitis virus (JEV, genus *Flavivirus*, family *Flaviviridae*). Two 870-nucleotides-long segments (YunnanJEV2017-1/2), one 1064-nucleotides-long segments (YunnanJEV2017-3) and four 2001-nucleotides-long segments (YunnanJEV2017-4/5/6/7) that all shared ~98% nt identity and shared ~95% and ~95–97% aa identity respectively with each other were isolated. BLASTN against the GenBank identified these segments as homologous to the C+prM, E and M+E genes of JEV sequence from Laos isolated in 2009, with at least 86, 95, and 96% nt identity and 83, 91, and 94% aa identity respectively. Phylogenetic analysis showed that the newly identified JEV genes all belonged to genotype I (Figure 7).

### Viral identification of japanese encephalitis virus

**Figure 8:**
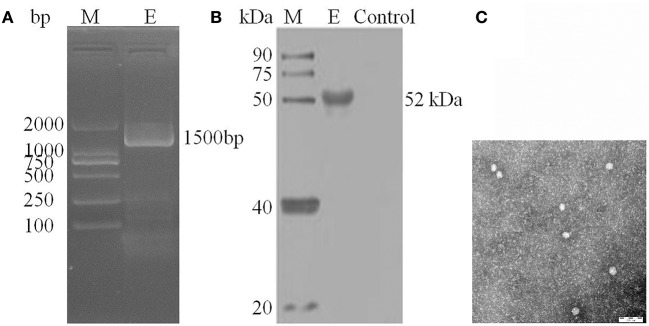
Identification of *JEV*-China/YN2017-4 isolation in Dali city of Yunnan province by PCR, Western blot, and negative-stain electron microscopy. PCR identification of *JEV*-China/YN2017-4 after BHK-21 cells infection **(A)**. Western blot identification of *JEV*-China/YN2017-4 isolation strain was assessed with an anti-E monoclonal antibody (Abcam, Cambridge, UK) and a HRP-conjugated goat anti-mouse antibody (ZSGB-Bio, Beijing, China) **(B)**. Negative-stain electron microscopy of *JEV*-China/YN2017-4 particles **(C)**.

After inoculation into BHK-21 cells, the positive JEV strain was verified by PCR technique. Levels of intracellular JEV were measured by Western blot method using an E-specific monoclonal antibody. YunnanJEV2017-4-infected cells were positive for E protein expression. Viral particles were observed using negative-stain electron microscopy. The particles of YunnanJEV2017-4 were rounded with a diameter of approximately 30–40 nm. Moreover, there were tiny protrusions on the surface, which appeared similar to JEV (Figure 8).

### The viral titers of the newly isolated JEV in cells

In BHK-21 cells, the mean viral titers of YunnanJEV2017-4 of different passage viruses were 5.36 × 10^4^ TCID_50_/0.1 mL (P5) and 6.42 × 10^4^ TCID_50_/0.1 mL (P10), respectively. It implied that multiple passages might result in various viral titers The YunnanJEV2017-4 E genes of P5 and P10 were aligned using MegAlign after sequencing. Nucleotide identity analysis showed that P5 shared 98.9% nt identity with P10. After passage in Vero cells using YunnanJEV2017-4 from infected BHK-21 cells, the E genes of YunnanJEV2017-4 (P5 and P10) from BHK-21 and Vero cells were sequenced. By nucleotide alignment, 11 and 12 nucleotide sites in E genes of JEV (P5 and P10) appeared polymorphic respectively. By means of amino acid sequences alignment, one amino acid site (position: 138 aa) in E proteins of P5 and P10 was both mutated, with amino acid lysine (K) changing to glutamic (E). The viral titers were detected in Vero cells in triplicate, and mean of those of YunnanJEV2017-4 were 2.84 × 10^5^ TCID_50_/0.1 mL (P5) and 3.57 × 10^5^ TCID_50_/0.1 mL (P10), respectively. It showed a significant increase (*P* < 0.05) compared to BHK-21 cells.

## Discussion

Mosquitoes are the intermediate host of numerous viruses that infect humans, animals, insects, plants, and other species, and play a significant role in the prevalence of many infectious diseases (Zhang et al., 2013; Fisher et al., 2015; Shi et al., 2015). Virus identification by traditional virological methods was very laborious and time-consuming before 2007 (Krah, 1991). Compared with the traditional method, metagenomic sequencing by Illumina sequencing cooperated with high throughput analysis is more efficiency, resulting in identification of many viruses from different species in recent years by several laboratories (Wilson et al., 2015). Moreover, metagenomic sequencing can reveal the composition of the virome in the mosquito samples. Metagenomic sequencing revealed the high abundance of viruses in mosquitoes (Bolling et al., 2015; Joseph et al., 2016; Shi et al., 2017). It can also contributed to the discovery of novel viruses and their identification and characterization (Carissimo et al., 2016; Frey et al., 2016). In the present study, more than 50 viral families were detected by Illumina sequencing in mosquito samples. However, there was a difference in the percentage of viral sequences obtained from the three mosquito groups. It could be related to the geographic location of the vectors (Atoni et al., 2018; Xia et al., 2018; Zakrzewski et al., 2018). Interestingly, the results show that these (and many other) viruses can be (collectively) found in non-Ae. aegypti mosquito species (Ae. albopictus was included in the samples) (Dodson and Rasgon, 2017; Dodson et al., 2018). Moreover, new findings of viral distribution and evolution in Yunnan province, China were obtained.

RT-PCR and PCR were used in confirming the results of Illumina sequencing. The detection of DENV in the mosquito samples shows that DENV still exist in Yunnan province, China. Many strains of DENV were uncovered by previous studies (Shi et al., 2014). The detection of new DENV strains is important, as it suggests a new prevalence of DENV in this area. In addition, the identification of novel circovirus is significant, suggesting that increased environmental surveillance is required because of its severe infectivity to animals, especially pigs, which are significant food animals. Meanwhile, the presence of unidentified viruses in the Illumina sequencing results may be ascribed to inadequate mosquito sampling and limited collection sites.

JEV and DENV were detected simultaneously, indicating that these viruses co-circulate in Yunnan province and implying that co-infection by the viruses is possible. Though the detection of the virus is not sufficient to prove transmission, detection of co-circulation of DENV and JEV in the same geographic location is important (Dodson and Rasgon, 2017; Dodson et al., 2018). The simultaneous detection of the genes of two viruses may bring a new challenge to the control of DENV.

The detection and isolation of JEV indicate that the virus still exists in Yunnan province. After passage in Vero cells using YunnanJEV2017-4 from infected BHK-21 cells, one amino acid site (position: 138 aa) in E proteins of JEV (P5 and P10) was both mutated, with lysine changing to glutamic. It was reported that, at the position of 138 in the E protein, lysine in that could attenuate viral infectivity, whereas glutamic could enhance viral infectivity (Ni et al., 1994). Subsequently, the viral titers were detected, and we found that there was an increase compared to BHK cells. The results show that viral titer of YunnanJEV2017-4 increased when passaged in Vero cells, suggesting the possible increased threat to human health induced by JEV transmission crossing species barrier.

Clearly, our results show that the distribution of viruses in mosquitoes collected in Yunnan province is dependent on the geographical location of mosquitoes. However, the present work explored only a small portion of the virome of mosquitoes in this region. More study in different areas and countries might be needed to assess the diversity of mosquito-borne viruses. In conclusion, our findings provide a useful insight into viral isolation and characterization of DENV and novel circovirus, which could be applied in the future.

## Author contributions

PX, HJL, and NJ conceived and designed the experiments. PX, JH, CL, YL, MW, and NJ performed the experiments. PX, HL, JR, and NJ analyzed the data. YZ, XG, LX, HZ, HL, and MT contributed reagents, materials, analysis tools. PX and NJ wrote the paper. All authors read and approved the final version of the manuscript.

### Conflict of interest statement

The authors declare that the research was conducted in the absence of any commercial or financial relationships that could be construed as a potential conflict of interest.
