# Supplementary material for: Metagenomic Sequencing From Mosquitoes in China Reveals a Variety of Insect and Human Viruses
Source: Front Cell Infect Microbiol. 2018 Oct 19;8:364. doi: 10.3389/fcimb.2018.00364 (PMC6202873; doi:10.3389/fcimb.2018.00364)
Supplement: Supplementary file 1 [file Table_1.DOCX]

**Table S1.** **The profile of mosquito samples employed in metagenomic analysis.**

| **Sample** | **Species** | **Number** | **Total Number** | **Location** | **GPS coordinates** |
| --- | --- | --- | --- | --- | --- |
| Sample I | *Culex tritaeniorhynchus* | 296 | 1000 | Ninger County | N 23°03’, E 101°02’ |
|  | *Armigeres obturbans* | 327 |  |  |  |
|  | *Aedes albopictus* | 44 |  |  |  |
|  | *Anopheles sinensis* | 23 |  |  |  |
|  | *Anopheles maculatus* | 100 |  |  |  |
|  | *Anopheles mininus* | 33 |  |  |  |
|  | *Culex quinquefasciatus* | 177 |  |  |  |
| Sample II | *Culex tritaeniorhynchus* | 233 | 1000 | Jinggu County | N 23°50′, E 100°71′ |
|  | *Armigeres obturbans* | 350 |  |  |  |
|  | *Aedes albopictus* | 122 |  |  |  |
|  | *Anopheles maculatus* | 82 |  |  |  |
|  | *Anopheles mininus* | 129 |  |  |  |
|  | *Culex quinquefasciatus* | 84 |  |  |  |
| Sample III | *Culex tritaeniorhynchus* | 6231 | 6700 | Dali City | N 25°69′, E 100°19′ |
|  | *Aedes albopictus* | 469 |  |  |  |
| Total |  |  | 8700 |  |  |

**Table S2. The reads number of viral families in metagenomic analysis of three mosquito samples.**

| Viral families | Sample I | Sample II | Sample III |
| --- | --- | --- | --- |
| Luteoviridae | 1406 | 283 | 248 |
| Marseilleviridae | 337 | 8 | 21 |
| Tombusviridae | 413 | 5 | 46 |
| Parvoviridae | 275055 | 8 | 196 |
| Adenoviridae | 5 | 16 | 26 |
| Baculoviridae | 167 | 23 | 84 |
| Nudiviridae | 3 | 1 | 4 |
| Podoviridae | 514 | 72 | 287 |
| Reoviridae | 14 | 443 | 383 |
| Secoviridae | 1 | 18 | 9 |
| Bunyaviridae | 5406 | 1901 | 441 |
| Iridoviridae | 4 | 10 | 154 |
| Iflaviridae | 48568 | 8019 | 23 |
| Dicistroviridae | 18 | 1 | 45 |
| Ascoviridae | 1 | 6 | 2 |
| Inoviridae | 14 | 26 | 22 |
| Orthomyxoviridae | 19 | 2 | 4 |
| Asfarviridae | 1 | 3 | 1 |
| Caulimoviridae | 47 | 2 | 37 |
| Nodaviridae | 450 | 10157 | 995 |
| Herpesviridae | 2 | 7 | 2 |
| Arteriviridae | 3668 | 3 | 221 |
| Circoviridae | 465 | 288 | 95 |
| Rhabdoviridae | 6543 | 1569 | 152 |
| Mimiviridae | 518 | 173 | 269 |
| Closteroviridae | 5 | 120 | 189 |
| Polydnaviridae | 450 | 50 | 109 |
| Phycodnaviridae | 2019 | 123 | 376 |
| Picornaviridae | 1181 | 7 | 96 |
| Partitiviridae | 1422 | 9147 | 501 |
| Retroviridae | 90 | 139 | 159 |
| Myoviridae | 1899 | 495 | 1186 |
| Nyamiviridae | 2 | 1 | 11 |
| Mesoniviridae | 95 | 6 | 116 |
| Permutotetraviridae | 60 | 29 | 68 |
| Siphoviridae | 1347 | 632 | 857 |
| Flaviviridae | 46669 | 2280 | 723423 |
| Tymoviridae | 44 | 232 | 119 |
